# Pembrolizumab in Chinese patients with advanced melanoma: 3-year follow-up of the KEYNOTE-151 study

**DOI:** 10.3389/fimmu.2022.882471

**Published:** 2022-10-11

**Authors:** Lu Si, Xiaoshi Zhang, Yongqian Shu, Hongming Pan, Di Wu, Jiwei Liu, Lili Mao, Xuan Wang, Xizhi Wen, Yanhong Gu, Lingjun Zhu, Shijie Lan, Xin Cai, Scott J. Diede, Haiyan Dai, Cuizhen Niu, Jianfeng Li, Jun Guo

**Affiliations:** ^1^ Key Laboratory of Carcinogenesis and Translational Research, Ministry of Education/Beijing, Peking University Cancer Hospital and Institute, Beijing, China; ^2^ Sun Yat-sen University Cancer Centre, Guangzhou, China; ^3^ Jiangsu Province Hospital, Nanjing, China; ^4^ Sir Run Run Shaw Hospital, Zhejiang University School of Medicine, Hangzhou, China; ^5^ The First Hospital of Jilin University, Changchun, China; ^6^ The First Affiliated Hospital of Dalian Medical University, Dalian, China; ^7^ Merck and Co., Inc., Rahway, NJ, United States; ^8^ MSD (China) Co., Ltd., Shanghai, China; ^9^ MSD (China) Co., Ltd., Beijing, China

**Keywords:** pembrolizumab, PD-1, PD-L1, advanced melanoma, cutaneous acral melanoma, mucosal melanoma

## Abstract

Survival is generally poor for Chinese patients with advanced melanoma because of high rates of acral and mucosal melanoma and limited therapeutic options. The first analysis of the phase 1b KEYNOTE-151 study showed second-line pembrolizumab was well tolerated and had clinically meaningful antitumor activity in Chinese patients with advanced melanoma. Three-year follow-up is presented. Eligible patients were of Chinese descent and had unresectable stage III/IV melanoma that progressed after first-line therapy. Patients received pembrolizumab 2 mg/kg every 3 weeks for ≤35 cycles. Primary end points were safety and objective response rate (ORR). Secondary end points included duration of response (DOR), progression-free survival (PFS), and overall survival (OS). Response was assessed per RECIST v1.1 by blinded independent central review. Subgroup analyses were conducted by melanoma subtype and *BRAF* and PD-L1 status (acral melanoma only). 103 patients were enrolled; median follow-up duration (time from first dose to data cutoff [July 13, 2020]) was 44.6 months (IQR, 39.1–46.2). Any-grade treatment-related adverse events (TRAEs) occurred in 85.4% of patients, and grade 3/4 TRAEs in 12.6%. No grade 5 TRAEs occurred. Three patients discontinued pembrolizumab because of TRAEs (immune-mediated hepatitis, pneumonia, and arthritis). Immune-mediated AEs and infusion reactions occurred in 34.0% (grade 3/4, 2.9%). ORR was 17.6% (95% CI, 10.8–26.4; 1 complete response/17 partial responses), and median DOR was 13.8 months (range, 2.7–37.4+). Median PFS was 2.8 months (95% CI, 2.7–3.5) and 36-month PFS rate was 5.0%. Median OS was 13.2 months (95% CI, 10.4–16.5) and 36-month OS rate was 22.3%. Median OS for patients with known melanoma subtype was 14.8 months for acral, 13.5 months for nonacral cutaneous, and 7.4 months for mucosal melanoma. Among the acral subgroup, median OS was 22.8 months for PD-L1–positive disease, 8.4 months for PD-L1–negative disease, 18.5 months for *BRAF* wild-type disease, and 5.8 months for *BRAF*-mutant disease. Over 3 years’ follow-up, second-line pembrolizumab continued to show manageable safety, clinically meaningful antitumor activity, and durable responses in Chinese patients with advanced melanoma. Subgroup analysis suggested particular benefit in PD-L1–positive and *BRAF* wild-type acral melanoma, although small subgroup sizes preclude definitive conclusions.

**Clinical trial registration:**

https://clinicaltrials.gov, identifier NCT02821000.

## 1 Introduction

Melanoma is a relatively rare disease in China, but the incidence is increasing rapidly (1). Although limited epidemiologic data are available, acral and mucosal melanomas are generally considered the most common subtypes in Chinese patients (2). These tend to arise in regions shielded from solar ultraviolet exposure and are regarded as more aggressive than other types of melanoma (3). An analysis of 522 patients with malignant melanoma at a center in China reported that patients had a poor prognosis, which likely reflects the limited effective treatment options available (2, 4). Data from a real-world analysis conducted at the Beijing Cancer Hospital indicate that dacarbazine-based regimens are likely the most frequently used first-line therapies in China and that paclitaxel-platinum combinations were likely the most commonly used in the second line until the approval of the PD-1 inhibitors pembrolizumab and toripalimab as second-line treatment of melanoma in China in 2018 (5–7). Median overall survival (OS) with the chemotherapy-based regimens is poor, at 10.5 months with first-line therapy and 7.5 months with second-line therapy (5). Although immune checkpoint inhibitors have dramatically improved survival outcomes in White populations and are approved for use in China, there are limited available data regarding the safety and efficacy of immune checkpoint inhibitors in Chinese populations (4).

The phase 1b KEYNOTE-151 study (NCT02821000) evaluated the efficacy and safety of the PD-1 inhibitor pembrolizumab as second-line therapy in Chinese patients with advanced melanoma (8). At median follow-up of 7.9 months, pembrolizumab was shown to be well tolerated and had clinically meaningful and durable antitumor activity, including in patients with acral melanoma and mucosal melanoma. The objective response rate (ORR) among all patients was 16.7% (95% CI, 10.0–25.3), and the duration of response (DOR) was 8.4 months (range, 1.1+ to 11.0+ months) (8). Although these results are promising, the duration of follow-up was limited, and the long-term impact of pembrolizumab in Chinese patients remains unknown. Here, we present 3-year follow-up data for patients in the KEYNOTE-151 study, including subgroup analyses by melanoma subtype and by PD-L1 and *BRAF* mutation status in patients with acral melanoma.

## 2 Materials and methods

### 2.1 Study design and participants

KEYNOTE-151 was a multicenter, open-label, single-arm, phase 1b trial that investigated the efficacy and safety of pembrolizumab in Chinese patients with unresectable stage III or IV melanoma that had progressed after first-line chemotherapy or targeted therapy (excluding adjuvant or neoadjuvant therapy). Eligible patients were ≥18 years of age; of Chinese descent, born in China, and had a Chinese home address; had ≥1 measurable lesion per Response Evaluation Criteria in Solid Tumours, version 1.1 (RECIST v1.1); had an Eastern Cooperative Oncology Group (ECOG) performance status of 0 or 1; had provided a tumor sample for PD-L1 analysis; and had known *BRAF* mutation status or were willing to provide a tumor sample for *BRAF* genotyping. Patients with uveal or ocular melanoma were excluded, as were those who had received prior anti–PD-1, anti–PD-L1, or anti–PD-L2 therapy. Detailed methods have been described previously and the protocol is available online (8).

The study protocol and all amendments were approved by the relevant institutional review board or independent ethics committee at each site (**Supplementary Table 1**). The trial was conducted in accordance with the protocol and its amendments, Good Clinical Practice guidelines, and the provisions outlined in the Declaration of Helsinki. All patients provided written informed consent.

### 2.2 Procedures

Patients received intravenous pembrolizumab 2 mg/kg every 3 weeks for up to 35 cycles (approximately 2 years) or until disease progression, unacceptable toxicity, or patient or physician decision to discontinue treatment. Patients who developed investigator-determined radiographic disease progression after stopping treatment with pembrolizumab could receive a second course of pembrolizumab of up to 17 cycles. Eligible patients included those who stopped initial treatment with complete response (CR), had received ≥8 cycles of pembrolizumab, and ≥2 doses after initial CR was declared; or patients who had stable disease (SD) or better and discontinued treatment for reasons other than disease progression or intolerability after having received 35 cycles of pembrolizumab. Imaging by computed tomography or magnetic resonance imaging was performed at baseline and week 12, then every 6 weeks until week 48, and every 12 weeks thereafter. Response was assessed per RECIST v1.1 by blinded independent central review (BICR). Patients were followed for survival every 12 weeks after confirmed disease progression or start of new anticancer therapy. Adverse events (AEs) were monitored throughout the study and for 30 days after pembrolizumab discontinuation (90 days for serious AEs or AEs of clinical interest). AEs were graded according to the National Cancer Institute Common Terminology Criteria for Adverse Events, version 4.0. PD-L1 expression was assessed centrally in archival or newly obtained tumor tissue samples using PD-L1 IHC 22C3 pharmDx (Agilent). PD-L1 positivity was defined as staining of ≥1% of tumor cells or mononuclear inflammatory cells within or contiguous to nests of tumor cells.

### 2.3 Outcomes

Primary end points were safety and tolerability and ORR per RECIST v1.1 by BICR. Secondary end points included DOR and progression-free survival (PFS) per RECIST v1.1 by BICR, and OS.

### 2.4 Statistical assessments

*Post hoc* analysis of the efficacy and safety of pembrolizumab with 3 years of follow-up is presented. Safety and OS were assessed in all patients who received ≥1 dose of study treatment (all subjects as treated [ASaT] population). ORR and PFS were assessed in all patients who received ≥1 dose of study treatment and had baseline data (full analysis set [FAS] population). DOR was assessed in all patients who had a response. Efficacy was assessed by melanoma subtype: acral, nonacral cutaneous, and mucosal melanoma, and in patients whose melanoma subtype was unknown. Additional subgroup analyses by *BRAF* mutation status (wild type *vs* mutant) and PD-L1 status (positive *vs* negative) were conducted for patients with acral melanoma. The ORR point estimate and 95% CI were calculated using the exact binomial method. PFS, DOR, and OS were estimated using the Kaplan-Meier method. All statistical analyses were performed using SAS version 9.4.

## 3 Results

### 3.1 Patient characteristics

A total of 103 patients were enrolled in KEYNOTE-151; all received ≥1 dose of study treatment, and 102 patients had available baseline data. Baseline characteristics have been reported in detail previously (8). Median age was 52 years (range, 22–77), 57.3% (n = 59) of patients were female, 56.3% (n = 58) had an ECOG performance status of 1, 53.4% (n = 55) had stage M1c disease, 25.2% (n = 26) had liver metastases, 51.5% (n = 53) had PD-L1-positive disease, and 19.4% (n = 20) had *BRAF*-mutant disease. Most patients (77.7%; n = 80) had cutaneous melanoma (39.8% [n = 41] nonacral; 37.9% [n = 39] acral), 14.6% (n = 15) had mucosal melanoma, and melanoma subtype was unknown for 7.8% (n = 8). Median follow-up duration (defined as time from first dose to data cutoff [July 13, 2020]) was 44.6 months (interquartile range, 39.1–46.2). Of 103 patients treated, 14 (13.6%) completed treatment and 89 (86.4%) discontinued, mostly because of progressive disease (71.8%; n = 74) (**Supplementary Figure 1**).

### 3.2 Safety

The safety profile remained similar to that described previously. Treatment-related AEs (TRAEs) of any grade were reported in 85.4% (n = 88) of patients (**Table 1**). The most common were hypothyroidism (26.2%; n = 27), increased alanine aminotransferase level (23.3%; n = 24), and hypertriglyceridemia (22.3%; n = 23). Grade 3/4 TRAEs were reported in 12.6% (n = 13) of patients and included increased gamma-glutamyltransferase level (1.9%; n = 2) and neutropenia, fatigue, immune-mediated hepatitis, pneumonia, decreased platelet count, increased white blood cell count, hyponatremia, hypophosphatemia, arthritis, rhabdomyolysis, and rash (all 1.0%; n = 1). Three patients (2.9%) discontinued pembrolizumab because of a TRAE (immune-mediated hepatitis, pneumonia, and arthritis: 1.0%; n = 1 each). No patients died because of a TRAE.

**Table 1:** TRAEs occurring in ≥5% of patients (ASaT population).

TRAEs, n (%)	All treated patients, N = 103	
	Any grade	Grade 3–5
Any	88 (85.4)	13 (12.6)
Blood and lymphatic system disorders		
Anemia	12 (11.7)	0 (0.0)
Endocrine disorders		
Hypothyroidism	27 (26.2)	0 (0.0)
Hyperthyroidism	6 (5.8)	0 (0.0)
General disorders and administration site conditions		
Fatigue	15 (14.6)	1 (1.0)
Asthenia	6 (5.8)	0 (0.0)
Investigations		
Alanine aminotransferase increased	24 (23.3)	0 (0.0)
Blood lactate dehydrogenase increased	17 (16.5)	0 (0.0)
Aspartate aminotransferase increased	14 (13.6)	0 (0.0)
Blood bilirubin increased	14 (13.6)	0 (0.0)
White blood cell count decreased	12 (11.7)	1 (1.0)
Blood creatine phosphokinase increased	10 (9.7)	0 (0.0)
Neutrophil count decreased	10 (9.7)	0 (0.0)
Bilirubin conjugated increased	9 (8.7)	0 (0.0)
Blood cholesterol increased	8 (7.8)	0 (0.0)
Blood glucose increased	7 (6.8)	0 (0.0)
Blood bilirubin unconjugated increased	6 (5.8)	0 (0.0)
Blood urea increased	6 (5.8)	0 (0.0)
Metabolism and nutrition disorders		
Hypertriglyceridemia	23 (22.3)	0 (0.0)
Hyperglycemia	11 (10.7)	0 (0.0)
Decreased appetite	9 (8.7)	0 (0.0)
Hyperuricemia	9 (8.7)	0 (0.0)
Musculoskeletal and connective tissue disorders		
Arthralgia	6 (5.8)	0 (0.0)
Skin and subcutaneous tissue disorders		
Rash	15 (14.6)	1 (1.0)
Vitiligo	15 (14.6)	0 (0.0)
Pruritus	13 (12.6)	0 (0.0)

ASaT, all subjects as treated; TRAEs, treatment-related adverse events.

Immune-related AEs and infusion reactions were reported in 34.0% (n = 35) of patients (**Table 2**). The most common were hypothyroidism (26.2%; n = 27), hyperthyroidism (5.8%; n = 6), and thyroiditis (2.9%; n = 3). Grade 3/4 immune-related AEs and infusion reactions occurred in 2.9% (n = 3) of patients, and included hepatitis, myositis, and severe skin reactions (1.0%; n = 1 each). One patient (1.0%) discontinued pembrolizumab because of immune-related hepatitis. No patients died because of an immune-related AE or infusion reaction.

**Table 2:** Immune-related AEs and infusion reactions in ≥1 patient (ASaT population).

Event, n (%)	All treated patients, N = 103	
	Any grade	Grade 3–5
Any event (≥1)	35 (34.0)	3 (2.9)
Hepatitis	1 (1.0)	1 (1.0)
Hyperthyroidism	6 (5.8)	0 (0.0)
Hypothyroidism	27 (26.2)	0 (0.0)
Infusion reactions	2 (1.9)	0 (0.0)
Myositis	1 (1.0)	1 (1.0)
Pneumonitis	2 (1.9)	0 (0.0)
Severe skin reaction	1 (1.0)	1 (1.0)
Thyroiditis	3 (2.9)	0 (0.0)

AEs, adverse events; ASaT, all subjects as treated.

### 3.3 Efficacy

**Figure 1:**
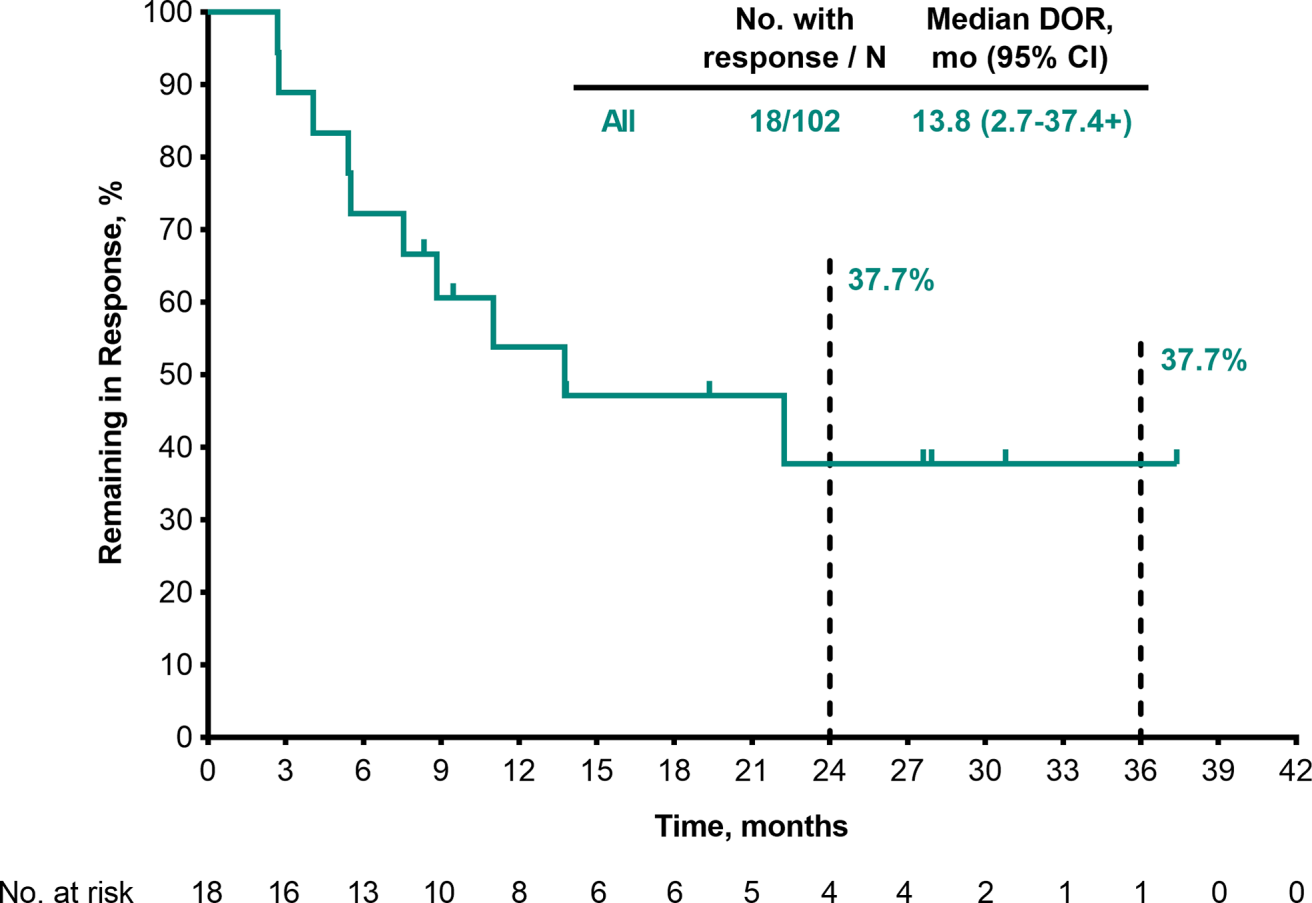
DOR per RECIST v1.1 by BICR (patients with response in the FAS population). BICR, blinded independent central review; DOR, duration of response; FAS, full analysis set; RECIST v1.1, Response Evaluation Criteria in Solid Tumours, version 1.1.

**Figure 2:**
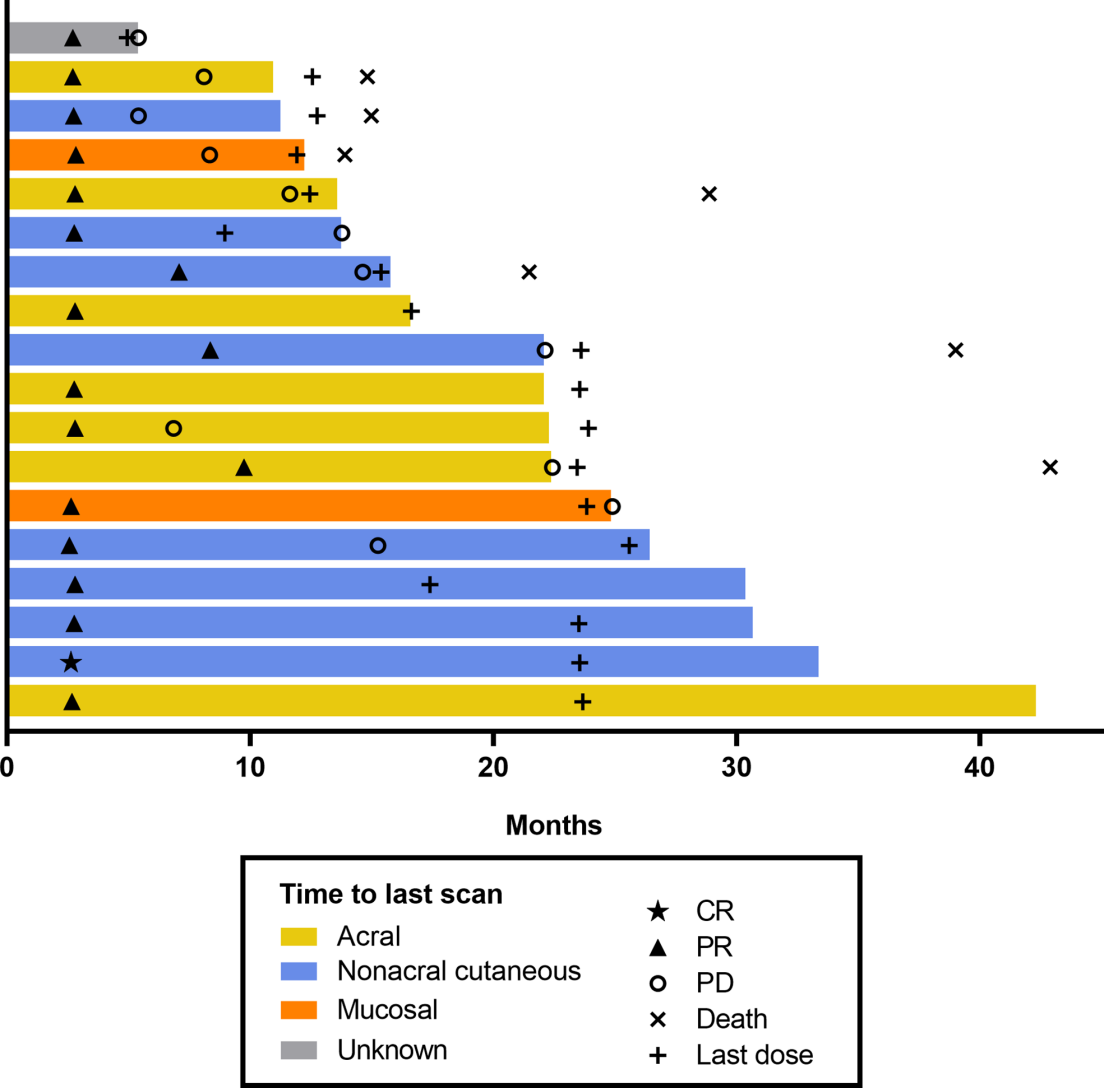
Swimmer plot of time to response per RECIST v1.1 by BICR (patients with response in the FAS population). BICR, blinded independent central review; CR, complete response; FAS, full analysis set; PD, progressive disease; PR, partial response; RECIST v1.1, Response Evaluation Criteria in Solid Tumours, version 1.1.

Among the 102 patients who were treated with pembrolizumab and had baseline data, ORR was 17.6% (95% CI, 10.8–26.4; 1 CR/17 partial response [PR]) and disease control rate (DCR) was 38.2% (95% CI, 28.8–48.4; 21 SD) (**Table 3**). Median DOR was 13.8 months (range, 2.7–37.4+) (**Table 3** **;** **Figure 1**), and 37.7% of patients had DOR ≥36 months. Median time to response was 2.8 months (range, 2.6–9.7) (**Figure 2**). One patient completed a second course of pembrolizumab. This patient had a best overall response of PR during the first course and achieved CR during the second course.

**Table 3:** Response characteristics per RECIST v1.1 by BICR.

Melanoma subtype	Subgroup	n^a^	ORR		DCR		DOR, median (range), months
			n	% (95% CI)	n	% (95% CI)	
Total	All	102	18	17.6 (10.8–26.4)	39	38.2 (28.8–48.4)	13.8 (2.7–37.4+)
Acral	All	38	7	18.4 (7.7–34.3)	16	42.1 (26.3–59.2)	NR (4.1–37.4+)
	PD-L1 positive	19	5	26.3 (9.1–51.2)	10	52.6 (28.9–75.6)	8.8 (4.1–13.8+)
	PD-L1 negative	16	2	12.5 (1.6–38.3)	5	31.3 (11.0–58.7)	NR (19.4+ to 37.4+)
	PD-L1 unknown	3	0	0.0 (0.0–70.8)	1	33.3 (0.8–90.6)	—
	*BRAF* wild-type	34	7	20.6 (8.7–37.9)	16	47.1 (29.8–64.9)	NR (4.1–37.4+)
	*BRAF* mutant	4	0	0.0 (0.0–60.2)	0	0.0 (0.0–60.2)	—
Nonacral cutaneous	All	41	8	19.5 (8.8–34.9)	17	41.5 (26.3–57.9)	13.8 (2.7–30.8+)
Mucosal	All	15	2	13.3 (1.7–40.5)	3	20.0 (4.3–48.1)	13.9 (5.5–22.2)
Unknown	All	8	1	12.5 (0.3–52.7)	3	37.5 (8.5–75.5)	2.7^b^

BICR, blinded independent central review; DCR, disease control rate; DOR, duration of response; FAS, full analysis set; NR, not reached; ORR, objective response rate; PD-L1, programmed death ligand 1; RECIST v1.1, Response Evaluation Criteria in Solid Tumours, version 1.1.

“+” indicates there is no progressive disease by the time of last disease assessment.

a In the FAS population.

b Duration of response in 1 patient.

**Figure 3:**
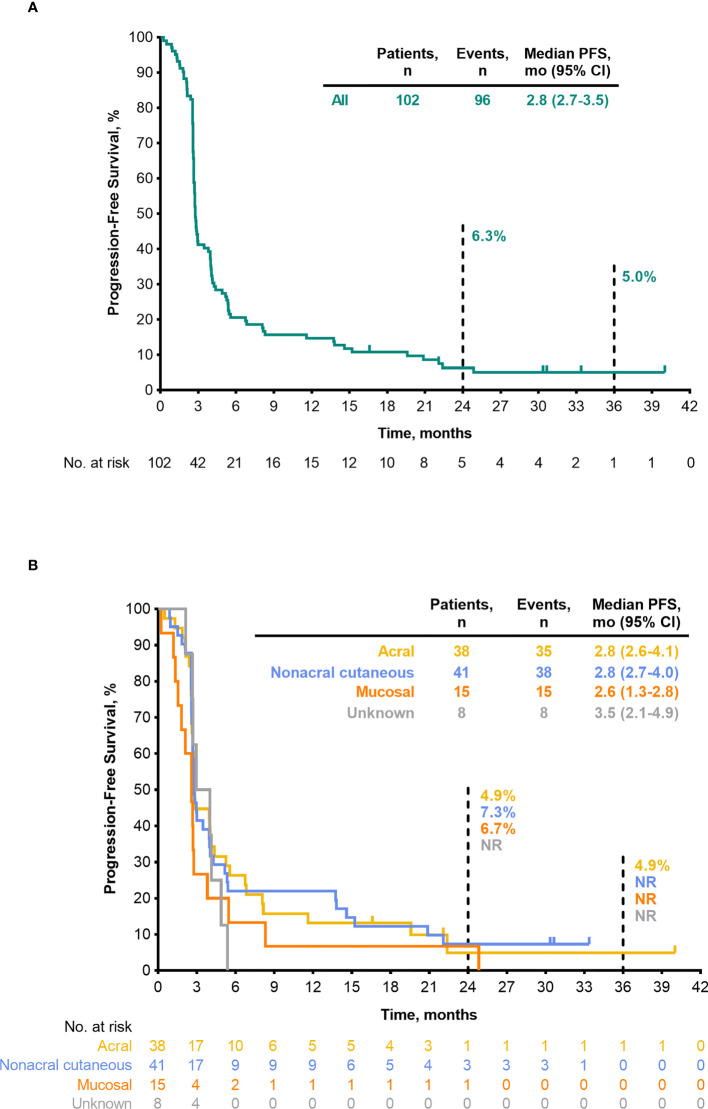
PFS per RECIST v1.1 by BICR (FAS population) **(A)** all patients **(B)** by melanoma subtype. BICR, blinded independent central review; FAS, full analysis set; NR, not reached; PFS, progression-free survival; RECIST v1.1, Response Evaluation Criteria in Solid Tumours, version 1.1.

**Figure 4:**
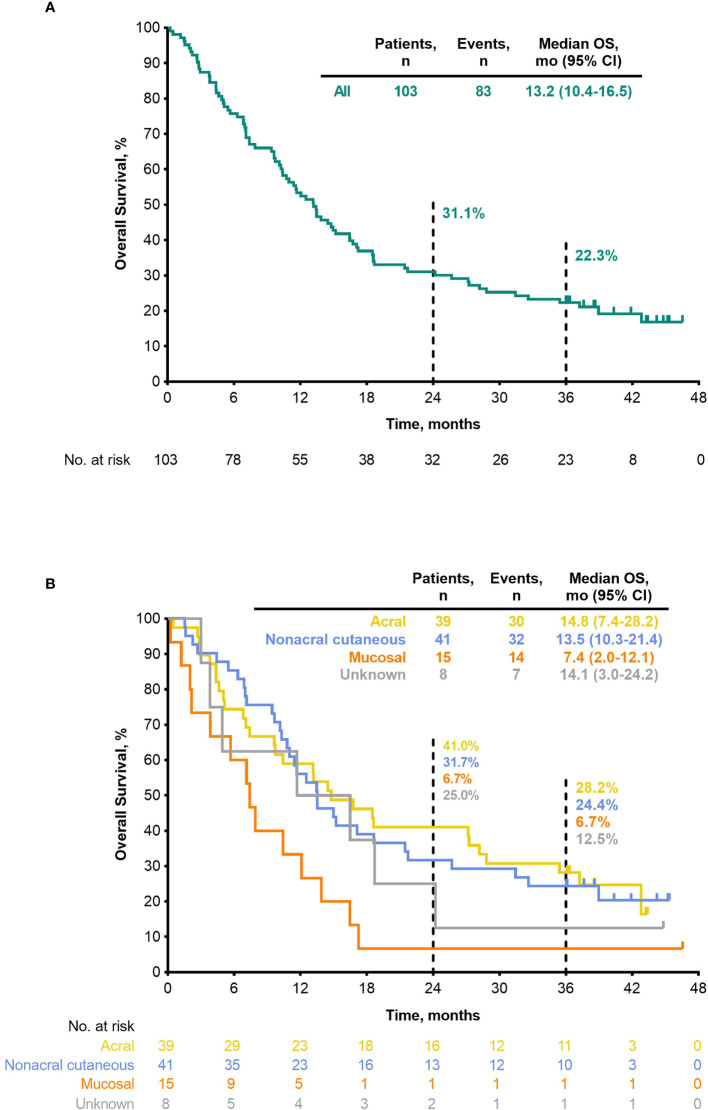
OS (ASaT population) **(A)** all patients **(B)** by melanoma subtype. ASaT, all subjects as treated; OS, overall survival.

**Figure 5:**
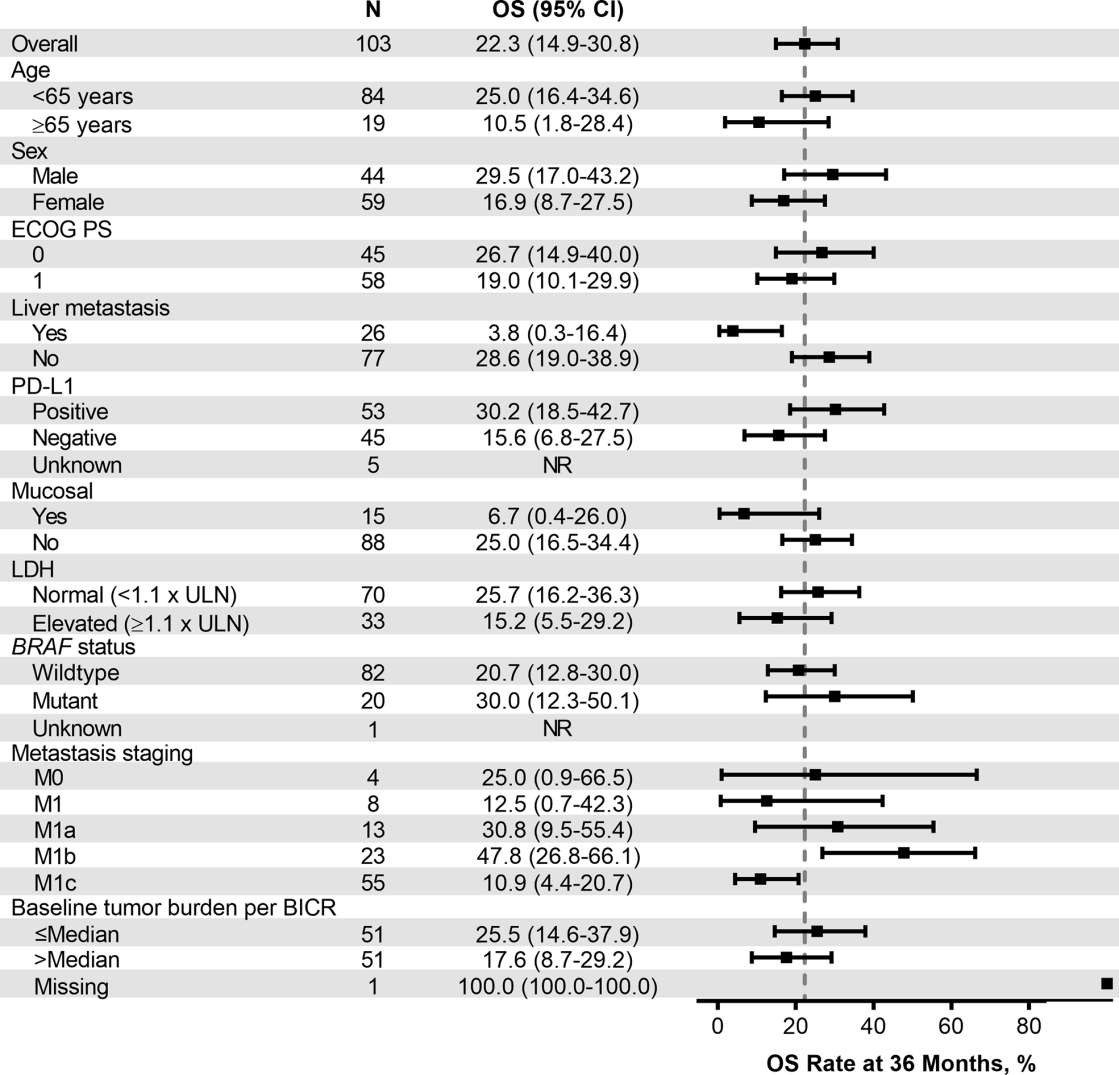
Forest plot of OS rate at 36 months in patient subgroups by baseline characteristics (ASaT population). ASaT, all subjects as treated; BICR, blinded independent central review; ECOG PS, Eastern Cooperative Oncology Group performance status; LDH, lactate dehydrogenase; OS, overall survival; PD-L1, programmed death ligand 1; ULN, upper limit of normal.

Median PFS was 2.8 months (95% CI, 2.7–3.5) (**Table 4**), the 24-month PFS rate was 6.3%, and the 36-month rate was 5.0% (**Figure 3A**). As of the data cutoff, 83 deaths had occurred. Median OS among all patients who received treatment was 13.2 months (95% CI, 10.4–16.5) (**Table 4**), the 24-month OS rate was 31.1%, and the 36-month rate was 22.3% (**Figure 4A**). In a subgroup analysis, 36-month OS rates were low for patients with liver metastases (3.8%) and patients with mucosal melanoma (6.7%) (**Figure 5**), and ORR was low for patients ≥65 years (5.6%) and patients with liver metastases (0.0%) (**Supplementary Figure 2** **)**. Twelve-month PFS rates were similar between subgroups (**Supplementary Figure 3**).

**Table 4:** PFS per RECIST v1.1 by BICR^a^ and OS^b^.

Melanoma Subtype	Subgroup	n^a^	PFS, median (95% CI),^c^ months	n^b^	OS, median (95% CI),^c^ months
Total	All	102	2.8 (2.7–3.5)	103	13.2 (10.4–16.5)
Acral	All	38	2.8 (2.6–4.1)	39	14.8 (7.4–28.2)
	PD-L1 positive	19	4.4 (2.7–6.8)	20	22.8 (7.4–37.2)
	PD-L1 negative	16	2.7 (2.6–4.0)	16	8.4 (4.6–28.2)
	PD-L1 unknown	3	2.6 (2.6–4.0)	3	13.1 (9.6–35.4)
	*BRAF* wild-type	34	3.4 (2.7–5.3)	35	18.5 (10.4–28.8)
	*BRAF* mutant	4	1.9 (0.5–2.6)	4	5.8 (0.5–7.4)
Nonacral cutaneous	All	41	2.8 (2.7–4.0)	41	13.5 (10.3–21.4)
Mucosal	All	15	2.6 (1.3–2.8)	15	7.4 (2.0–12.1)
Unknown	All	8	3.5 (2.1–4.9)	8	14.1 (3.0–24.2)

ASaT, all subjects as treated; BICR, blinded independent central review; FAS, full analysis set; OS, overall survival; PD-L1, programmed death ligand 1; PFS, progression-free survival; RECIST v1.1, Response Evaluation Criteria in Solid Tumours, version 1.1.

a In the FAS population.

b In the ASaT population.

c From product-limit (Kaplan-Meier) method for censored data.

#### 3.3.1 Efficacy by melanoma subtype

ORR by melanoma subtype was 18.4% (0 CR/7 PR) for patients with acral melanoma, 19.5% (1 CR/7 PR) for patients with nonacral cutaneous melanoma, 13.3% (0 CR/2 PR) for patients with mucosal melanoma, and 12.5% (0 CR/1 PR) for patients with unknown melanoma subtype (**Table 3**). Median DOR was not reached (NR; range, 4.1–37.4+ months), 13.8 months (range, 2.7–30.8+), and 13.9 months (range, 5.5–22.2), in patients with acral, nonacral cutaneous, and mucosal melanoma, respectively (**Table 3**). One patient with unknown melanoma subtype had a response duration of 2.7 months. Median PFS was 2.8 months in patients with acral melanoma, 2.8 months in patients with nonacral cutaneous melanoma, 2.6 months in patients with mucosal melanoma, and 3.5 months in patients with unknown melanoma subtype (**Table 4** **;** **Figure 3B**). Median OS was 14.8 months in patients with acral melanoma, 13.5 months in patients with nonacral cutaneous melanoma, 7.4 months in patients with mucosal melanoma, and 14.1 months in patients with unknown melanoma subtype (**Table 4** **;** **Figure 4B**).

In a subgroup analysis of patients with acral melanoma by PD-L1 status (PD-L1 positive, n = 19; PD-L1 negative, n = 16; PD-L1 unknown, n = 3), ORR was 26.3% (95% CI, 9.1–51.2; 0 CR/5 PR) in patients with PD-L1–positive disease, 12.5% (95% CI, 1.6–38.3; 0 CR/2 PR) in patients with PD-L1–negative disease, and 0% in the 3 patients with unknown PD-L1 status (**Table 3**). Median DOR was 8.8 months (range, 4.1–13.8+) in patients with PD-L1–positive acral melanoma and NR (range, 19.4+ to 37.4+ months) in patients with PD-L1–negative acral melanoma (**Table 3**). Median PFS was 4.4 months (95% CI, 2.7–6.8) in patients with PD-L1–positive disease, 2.7 months (95% CI, 2.6–4.0) in patients with PD-L1–negative disease, and 2.6 months (95% CI, 2.6–4.0) in patients with unknown PD-L1 status (**Table 4**). Median OS was 22.8 months (95% CI, 7.4–37.2), 8.4 months (95% CI, 4.6–28.2), and 13.1 months (95% CI, 9.6–35.4) in patients with PD-L1–positive disease, PD-L1–negative disease, and disease of unknown PD-L1 status, respectively (**Table 4**).

In a subgroup analysis of patients with acral melanoma by *BRAF* mutation status (*BRAF* wild type, n = 34; *BRAF* mutant, n = 4), ORR was 20.6% (95% CI, 8.7–37.9; 0 CR/7 PR) in patients with *BRAF* wild-type disease and 0% in the 4 patients with *BRAF*-mutant disease (**Table 3**). Median DOR was NR (range, 4.1–37.4+ months) in patients with *BRAF* wild-type disease (**Table 3**). Median PFS was 3.4 months (95% CI, 2.7–5.3) in patients with *BRAF* wild-type acral melanoma and 1.9 months (95% CI, 0.5–2.6) in patients with *BRAF*-mutant disease (**Table 4**). Median OS was 18.5 months (95% CI, 10.4–28.8) and 5.8 months (95% CI, 0.5–7.4) in patients with *BRAF* wild-type and *BRAF*-mutant acral melanoma, respectively (**Table 4**).

## 4 Discussion

In this 3-year follow-up of the KEYNOTE-151 study, second-line pembrolizumab had a manageable safety profile and continued to show clinically meaningful antitumor activity, durable responses, and prolonged survival in Chinese patients with advanced melanoma. The ORR of 17.6%, DCR of 38.2%, DOR of 13.8 months, and the 36-month OS rate of 22.3% also suggest that pembrolizumab has durable survival benefit in some patients.

The safety profile of pembrolizumab in the current study was similar to that observed at the earlier analysis of KEYNOTE-151 and was generally consistent with the known safety profile of pembrolizumab in global populations (8, 9). The only notable difference compared with the earlier analysis was a higher incidence of treatment-related alanine aminotransferase increase (23.3% *vs* 14.6%) and vitiligo (14.6% *vs <*2%). While the rate of vitiligo was similar to that observed in a global pooled analysis of patients with melanoma receiving pembrolizumab (12.0%), the number of patients reporting elevated alanine aminotransferase in KEYNOTE-151 was comparably much higher (4.4%) (9). Although it is unclear why the incidence of alanine aminotransferase increase was so high in this study, all occurrences were grade 1 or 2, indicating they were mild or moderate in severity. Of note, a high rate of treatment-related alanine aminotransferase increase (31.3%) was also observed in the phase 2 POLARIS-01 study, which investigated the PD-1 inhibitor toripalimab in 128 Chinese patients with advanced melanoma previously treated with systemic therapy (10). Elevated alanine aminotransferase levels may therefore be an AE that occurs more frequently with PD-1 inhibitors in patients of Chinese descent.

The efficacy of immune checkpoint inhibitors has been well established in White patients, but there are limited data available regarding their use in Chinese patients, or even the wider Asian population. To our knowledge, the only other prospective trial of an immune checkpoint inhibitor in Chinese patients with data available is the phase 2 POLARIS-01 study (10). POLARIS-01, which included 50 (39.4%) patients with acral melanoma and 22 (17.3%) patients with mucosal melanoma, reported an ORR of 17.3% (1 CR/21 PR), which was similar to that observed in the current analysis. The DCR was 57.5%, the DOR was NR, and the median PFS and OS were 3.6 months and 22.2 months, respectively. These results are suggestive of a greater magnitude of benefit than those observed in the current analysis; however, differences in study design and baseline characteristics, including a higher proportion of patients with stage IV disease and elevated lactate dehydrogenase levels in KEYNOTE-151, preclude direct comparison between the studies. Two retrospective studies have also been conducted. One study, which included 51 patients with melanoma treated with a regimen containing an anti–PD-1 antibody at a single center in China, reported an ORR of 10.8% and a median OS of 13.0 months in patients receiving PD-1 blockade alone (11). A retrospective case series of 52 Chinese patients with advanced melanoma who received immune checkpoint inhibitor therapy reported an ORR of 25.0% and a median OS of 10.0 months among patients who received pembrolizumab monotherapy (12). Although data regarding the efficacy of PD-1 inhibitors in Chinese patients are limited, several studies have been conducted in Japanese patients, who have comparably high rates of acral and mucosal melanoma (13). In the phase 1b KEYNOTE-041 study, at median follow-up of 10.3 months pembrolizumab was shown to have antitumor activity with an ORR of 24.3%, median DOR was NR, and median OS of NR in Japanese patients with unresectable stage III or advanced melanoma (14). Long-term follow-up of a phase 2 study investigating nivolumab as first-line therapy in Japanese patients with stage III/IV melanoma with a median follow-up of 32.9 months reported an ORR of 34.8% and a median OS of 32.9 months, although this analysis was limited by the small number of patients (n = 10) (15). Both the retrospective analyses of immune checkpoint inhibitors in Chinese patients and the results of the prospective study investigating pembrolizumab in Japanese patients reported outcomes that were generally comparable with those seen in the current analysis (ORR, 17.6%; median OS, 13.2 months), although differences in study design and patient populations preclude direct comparison.

As in the earlier analysis of the KEYNOTE-151 study (8), the efficacy of pembrolizumab was also investigated by melanoma subtype at the 3-year follow-up point. In this study, 37.9% of patients had acral melanoma and 14.6% had mucosal melanoma, which is comparable to the proportions observed in a pivotal study conducted in Chinese patients with melanoma (41.8% acral melanoma; 22.6% mucosal melanoma) (2). Data comparing outcomes with PD-1 inhibitors in Chinese patients with acral and mucosal melanoma are limited. In the POLARIS-01 study, the ORR was 14.0% for patients with acral melanoma and 0% for patients with mucosal melanoma (10). The median PFS was 3.2 months and 1.9 months, and the median OS was 16.9 months and 10.3 months, respectively. In retrospective analyses, ORRs with immune checkpoint inhibitors range from 18.8% to 26.7% for patients with acral melanoma and range from 17.6% to 20.0% for patients with mucosal melanoma (11, 12). In the current analysis, the efficacy of pembrolizumab was similar in acral and nonacral cutaneous melanoma (ORR, 18.4% and 19.5%, respectively; median OS, 14.8 months and 13.5 months, respectively). This differed from the results of the POLARIS-01 study, which reported better efficacy in patients with nonacral versus acral melanoma (ORR, 31.0% *vs* 14.0%; median OS, NR *vs* 16.9 months) (10). In the current analysis, patients with mucosal melanoma had relatively worse outcomes than patients with cutaneous melanoma subtypes, with a lower ORR (13.3%) and shorter median OS (7.4 months); note, however, the small number of patients in the mucosal subgroup (n = 15). These results are consistent with reports suggesting that patients with mucosal melanoma have unfavorable outcomes with immune checkpoint inhibitors (16, 17). A *post hoc* analysis of the KEYNOTE-001, KEYNOTE-002, and KEYNOTE-006 studies conducted in a global population reported an ORR of 19% with pembrolizumab in patients with mucosal melanoma and 33% in patients with nonmucosal melanoma, and median OS of 11.3 months and 23.5 months, respectively (16). A lower mutational burden, lower PD-L1 expression, and differences in tumor microenvironment are thought to contribute to the reduced efficacy of immune checkpoint inhibitors in mucosal melanoma (3). Although efficacy in the current analysis was lower in mucosal melanoma compared with cutaneous melanomas, pembrolizumab showed antitumor activity in both acral and mucosal melanoma.

Further subgroup analysis in patients with acral melanoma by PD-L1 expression and *BRAF* mutation status within the current analysis showed particularly good response and prolonged survival in patients with PD-L1–positive and *BRAF* wild-type disease. Although limited patient numbers in these subgroups preclude definitive conclusions, the observation warrants further investigation. Further subgroup analyses by PD-L1 and *BRAF* mutation status in other melanoma subtypes could not be conducted because of small sample sizes.

Although the current study is limited by the single-arm, open-label design, the results provide important data in a patient population with a significant unmet need for improved treatment options. The results of this *post hoc* exploratory analysis suggest that pembrolizumab as second-line therapy is well tolerated and provides clinically meaningful long-term antitumor activity in Chinese patients with advanced melanoma. Efficacy was observed across subgroups by PD-L1 expression and *BRAF* mutation status, with notable benefit in patients with acral melanoma who had PD-L1–positive or *BRAF* wild-type disease. These results strengthen the evidence showing that pembrolizumab is an appropriate second-line therapy for Chinese patients with advanced melanoma, regardless of subtype.

## Data availability statement

The original contributions presented in the study are included in the article/**Supplementary Material**. Further inquiries can be directed to the corresponding author.

## Ethics statement

The study protocol and all amendments were approved by the Ethics Committee of Beijing Cancer Hospital, The First Hospital of Jilin University First Affiliated Hospital of Dalian Medical University Ethics Committee, Sir Run Run Shaw Hospital, College of Medicine, Zhejiang University Ethics Committee of Sun Yat-Sen University Cancer Center Ethics Committee of Jiangsu Province Hospital. The trial was conducted in accordance with the protocol and its amendments, Good Clinical Practice guidelines, and the provisions outlined in the Declaration of Helsinki. The patients/participants provided their written informed consent to participate in this study.

## Author Contributions

LS, SD, JFL, and HD substantially contributed to the conception, design, or planning of the study. LS, XZ, YS, HP, DW, JWL, LM, XZW, YG, LZ, SL, XC, JFL, and JG substantially contributed to acquisition of data. LS and CN substantially contributed to analysis of the data. LS, XW, SD, JFL, and HD substantially contributed to interpretation of the results. LS and HD substantially contributed to drafting the manuscript. LS, XZ, YS, HP, DW, JWL, LM, XW, XZW, YG, LZ, SL, XC, SD, JFL, CN, and JG substantially contributed to critically reviewing or revising the manuscript for important intellectual content. All authors contributed to manuscript revision and approved the submitted version.

## Funding

This study was funded by Merck Sharp and Dohme LLC, a subsidiary of Merck and Co., Inc., Rahway, NJ, USA. The open access publication fees were also funded by the study sponsor.

## Acknowledgments

We thank the patients and their families and caregivers and all investigators and site personnel. Medical writing and/or editorial assistance was provided by Jemimah Walker, PhD, and Doyel Mitra, PhD, CMPP, of ApotheCom (Yardley, PA, USA). This assistance was funded by Merck Sharp and Dohme LLC, a subsidiary of Merck and Co., Inc., Rahway, NJ, USA.

## Conflict of interest

The study received funding from Merck Sharp and Dohme LLC, a subsidiary of Merck and Co., Inc., Rahway, NJ, USA. The funder had the following involvement with the study: the funder of the study collaborated with academic advisers in designing the study, gathering, analyzing, and interpreting the results, and payment of open access publication fees for the current manuscript. LS reports receiving honoraria from MSD, Roche, Juushi Bio, and Novartis. XW reports receiving grants to their institution from Oriengene. SD reports employment at Merck Sharp and Dohme LLC, a subsidiary of Merck and Co., Inc., Rahway, NJ, USA, and is a shareholder of Merck and Co., Inc., Rahway, NJ, USA. CN reports employment at MSD, China, and receiving honoraria from MSD, China. JFL reports employment at Merck Sharp and Dohme LLC, a subsidiary of Merck and Co., Inc., Rahway, NJ, USA. HD reports employment at MSD, China, and receiving honoraria from MSD, China. JG reports advisory/consultancy roles with MSD, Roche, Bayer, Novartis, Simcere Pharmaceutical Group, Shanghai Junshi Biosciences, and Oriengene.

The remaining authors declare that the research was conducted in the absence of any commercial or financial relationships that could be construed as a potential conflict of interest.

## Publisher’s note

All claims expressed in this article are solely those of the authors and do not necessarily represent those of their affiliated organizations, or those of the publisher, the editors and the reviewers. Any product that may be evaluated in this article, or claim that may be made by its manufacturer, is not guaranteed or endorsed by the publisher.

## Abbreviations

AEs, adverse events; ASaT, all subjects as treated; BICR, blinded independent central review; CR, complete response; DCR, disease control rate; DOR, duration of response; ECOG, Eastern Cooperative Oncology Group; FAS, full analysis set; OS, overall survival; ORR, objective response rate; PFS, progression-free survival; PD-L1, programmed death ligand 1; PR, partial response; RECIST v1.1, Response Evaluation Criteria in Solid Tumours, version 1.1; SD, stable disease; TRAEs, treatment-related adverse events.
