# Supplementary material for: Pembrolizumab in Chinese patients with advanced melanoma: 3-year follow-up of the KEYNOTE-151 study
Source: Front Immunol. 2022 Oct 11;13:882471. doi: 10.3389/fimmu.2022.882471 (PMC9593700; doi:10.3389/fimmu.2022.882471)
Supplement: Supplementary file 1 [file DataSheet_1.docx]

**SUPPLEMENTARY DATA**

**Supplementary Table 1 |** Ethics committees that approved the protocol and amendments at each site

| **Site No.** | **Name of ethics committees** |
| --- | --- |
| 0001 | Ethics Committee of Beijing Cancer Hospital |
| 0002 | Ethics Committee of The First Hospital of Jilin University |
| 0003 | First Affiliated Hospital of Dalian Medical University Ethics Committee |
| 0005 | Ethics Committee, Sir Run Run Shaw Hospital, College of Medicine, Zhejiang University |
| 0007 | Ethics Committee of Sun Yat-Sen University Cancer Center |
| 0010 | Ethics Committee of Jiangsu Province Hospital |

**Supplementary Figure 1 |** Trial profile

**
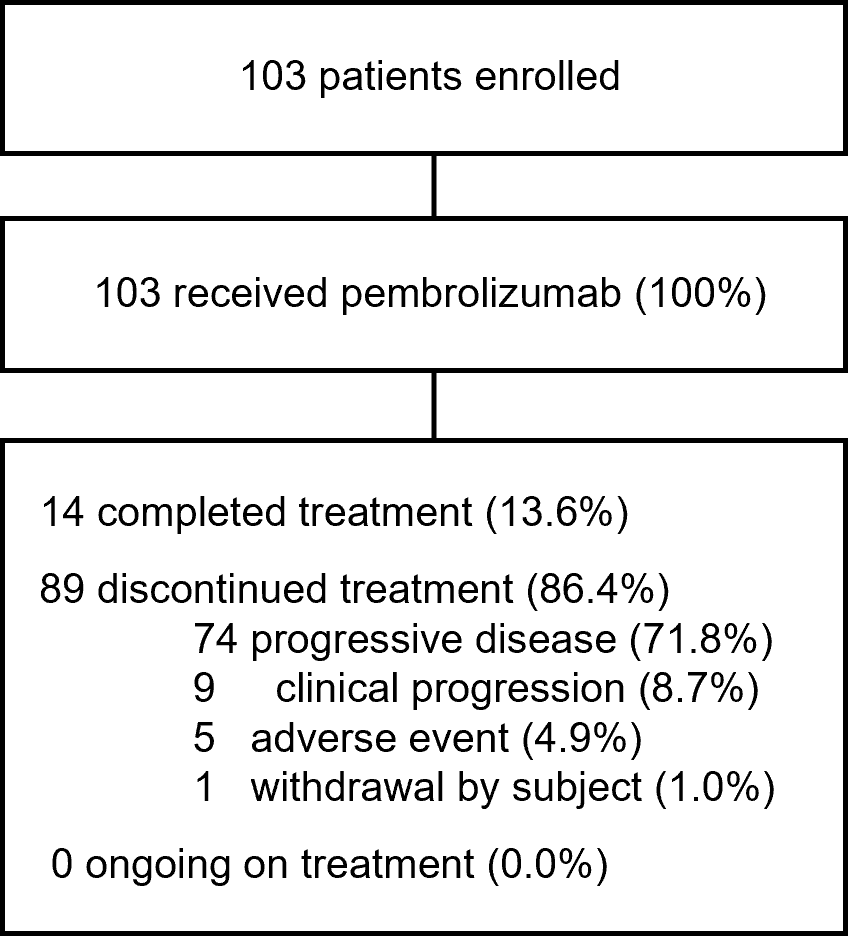
**

**Supplementary Figure 2 |** Forest plot of ORR (confirmed) per RECIST v1.1 by BICR (FAS population)


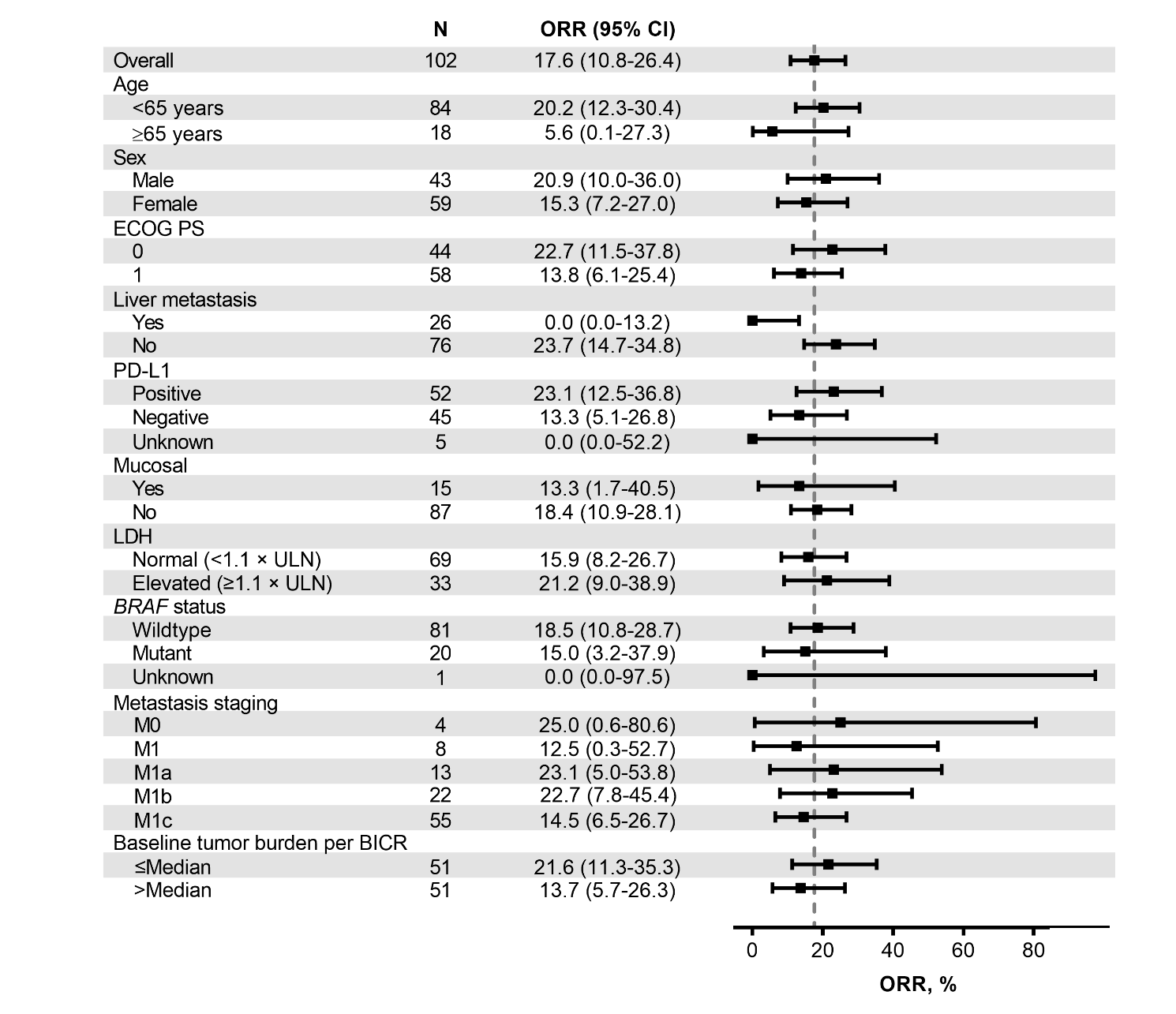


BICR, blinded independent central review; ECOG PS, Eastern Cooperative Oncology Group performance status; FAS, full analysis set; LDH, lactate dehydrogenase; ORR, objective response rate; PD-L1, programmed death ligand 1; RECIST v1.1, Response Evaluation Criteria in Solid Tumours, version 1.1; ULN, upper limit of normal.

**Supplementary Figure 3 |** Forest plot of PFS rate at 12 months per RECIST v1.1 by BICR (FAS population)


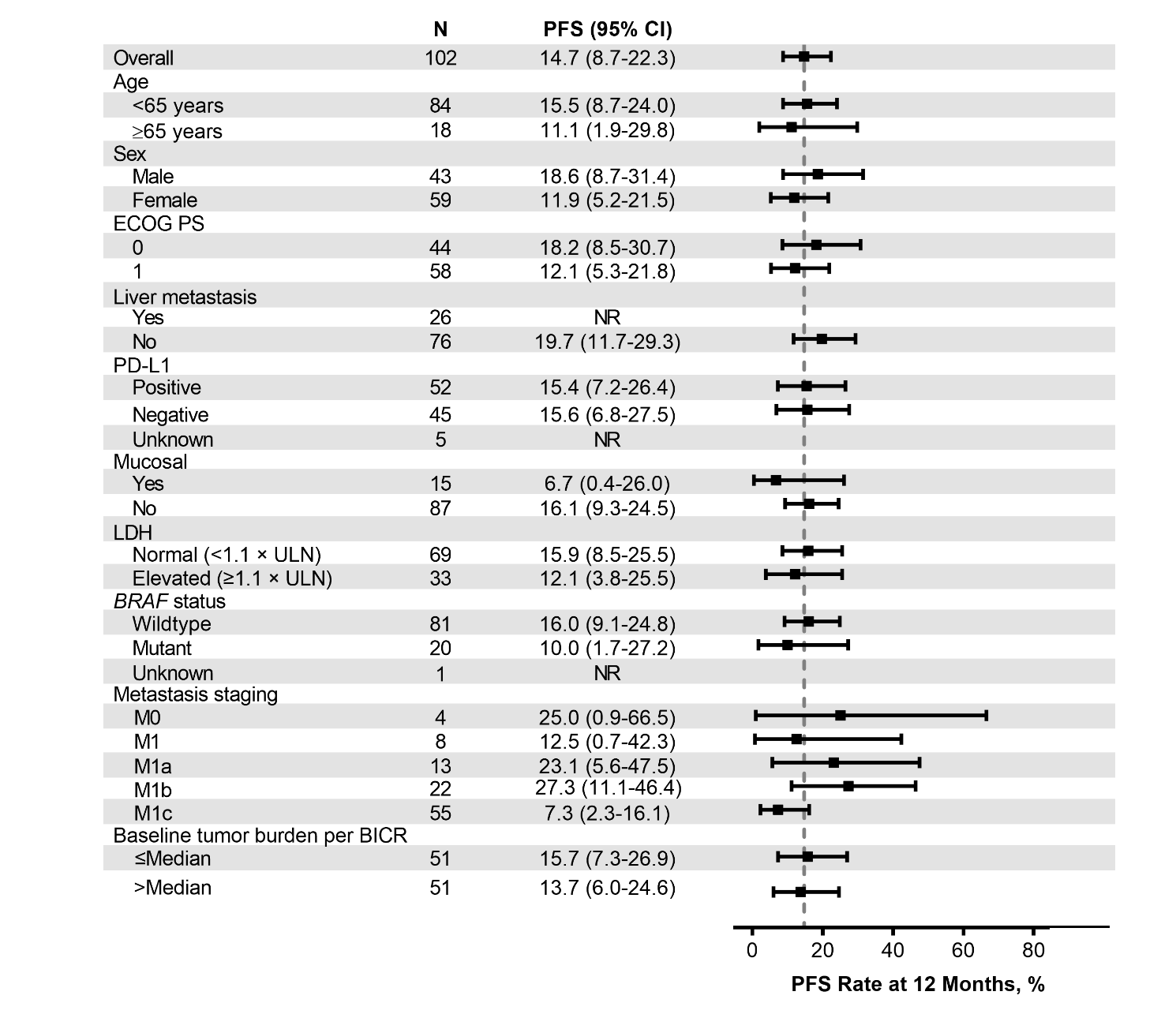


BICR, blinded independent central review; ECOG PS, Eastern Cooperative Oncology Group performance status; FAS, full analysis set; LDH, lactate dehydrogenase; PD-L1, programmed death ligand 1; PFS, progression-free survival; RECIST v1.1, Response Evaluation Criteria in Solid Tumours, version 1.1; ULN, upper limit of normal.
